# Editorial: Integrating multi-scale approaches for predicting microbiome ecology and evolution

**DOI:** 10.3389/fmicb.2022.1045045

**Published:** 2022-10-11

**Authors:** Andrea Quagliariello, Ricardo S. Ramiro, Alejandro Couce, Maria Elena Martino

**Affiliations:** ^1^Department of Comparative Biomedicine and Food Science, University of Padua, Padua, Italy; ^2^InnovPlantProtect, Elvas, Portugal; ^3^Centro de Biotecnología y Genómica de Plantas, Universidad Politécnica de Madrid (UPM), Madrid, Spain

**Keywords:** microbial ecology, microbial evolution, microbial communities, microbial adaptation, comparative genomics

An intricate balance of factors determines the evolution of microbes living in communities. Ever-changing environmental conditions and complex microbial interactions combine in non-linear ways to hamper the identification of the general principles and basic mechanisms governing the evolution of microbial communities (Little et al., 2008). However, recent work has started to disentangle the predominant factors underpinning these processes, mainly under stable laboratory conditions. In addition, a large body of research has been focusing on the development and integration of experimental and bioinformatic approaches toward the understanding of microbial evolution. The combination of meta-omics approaches, high-throughput sequencing, mathematical modeling and experimental approaches is shedding light into genetic mechanisms underlying strain- and species-specific adaptation within microbial communities evolving in complex and heterogeneous environments.

To illustrate these recent advances in the ‘Ecology and Evolution within Microbial Communities' field, we have selected nine articles for this Research Topic, which we believe focus on key avenues in this research field.

An important advancement in microbiome research has been the use of Experimental Evolution (EE), which largely facilitated the monitoring of bacterial genetic evolution and the understanding of adaptive evolution processes. EE corresponds to the study of the evolutionary modifications occurring within microbial populations in response to defined and controlled environmental stresses imposed by the experimenter (Kawecki et al., 2012; Barrick and Lenski, 2013; McDonald, 2019). Such experimental model makes it possible to constantly monitor the adaptation of populations to the surrounding environment by observing real-time evolution for ten, hundreds or even thousands of generations and allowing the detection of both phenotypic and genetic changes among individuals in the populations. Coupling EE with mass sequencing and comparative genomics allows us to unravel the genetic mechanisms underlying adaptation in bacteria at the genomic level. In this light, EE studies have strongly contributed to recognize the genetic changes that distinguish an evolved clone from its original ancestor strain in a wide range of experimental conditions and to reveal the functional networks involved in microbial adaptation (Segrè et al., 2006; Kawecki et al., 2012; Bailey and Bataillon, 2016). Historically, EE assays have mostly focused on low complexity systems, while few studies have been conducted on complex systems using natural microbial communities. The review by Manriquez et al.presents an outlook on microbial EE studies done with systems of increasing complexity (from single species, to synthetic communities and natural communities), with a particular focus on studies between plants and plant-associated microorganisms (e.g., focal bacterial pathogens adapting to different hosts; molecular mechanisms leading to microbe/plant beneficial interaction; bulk soil communities in response to artificial selection applied on plant phenotypes). In this regard, the authors point out the current need to distinguish to what extent plant phenotypic traits are determined by the environmental pressure and the microbial ecological and evolutionary dynamics.

One evolutionary advantage afforded by living in communities is the possibility of receiving useful external genes *via* horizontal gene transfer (HGT). However, the maintenance of mobile genetic elements in microbial communities depends on the delicate balance between the benefits and costs carrying these elements entails. The study by Cyriaque et al. challenges the simple expectation that the spreading success of plasmids carrying beneficial genes (in this case, heavy metal resistance genes—MRG) is greater in the presence of strong selective pressures in favor of those genes (i.e., high concentration of lead). Contrary to the expectations, the authors find that high concentrations of lead elicit physiological responses that inhibit conjugation and partition systems, thus reducing plasmid dispersal. Indeed, the spread of MRG-carrying plasmids was best at intermediate metal concentrations. Overall, this study paints a complex picture in which the success of mobile genetic elements hinges on the specific physiological and mutational propensities of the constituents of a community, suggesting that the opportunity for HGT-driven evolution may vary widely across microbial communities.

If the previous study shows that environmental stresses can translate into non-linear selective pressures, the study by Pinto et al.investigates how taking into consideration microbiome composition adds an extra layer of complexity to the genotype to fitness map. In particular, they studied the selective advantage constituted by the wellknown *lac* operon in *Escherichia coli* when colonizing the mammalian gut. By directly competing lac+ and lac– *E. coli* strains in the mouse gut, the authors found that, in the presence of lactose, the *lac* operon confers a selective advantage that can be as high as 11%, while being close to neutral in the absence of this sugar. Such fitness advantage directly depends on microbiota composition and on population density. In particular, as the number of species increases, so does the opportunity for competition (i.e., the ability to consume lactose represents an advantage as other nutrients get depleted) as well as opportunity for synergism, with the absolute success of *E. coli* reflecting the net result between these two forms of ecological interactions. In this regard, the authors found that the population size of *E. coli* substantially increases in the triple-colonization, in comparison to mono-colonization, suggesting that, in this scenario, synergism dominates. Conversely, in the presence of a complex microbiota (several tens of species), its absolute success decreases and the most likely scenario is one dominated by competition. The adaptive value of specific operons is thus primarily dependent on the microbial diversity of a given community and the specificity of interactions among species. Yet, the extent to which interactions with coexisting species can alter microbial evolution and diversification remains elusive. Hypotheses range from coexistence impeding diversification due to niche exclusion, to coexistence boosting diversification due to niche construction

On this subject, the work by Chu et al. documents one example of niche exclusion: the presence of multiple species reduces the evolutionary success of the dominant morphotype that takes over *Pseudomonas fluorescens* populations in a classic model of adaptive radiation. In particular, the authors show that the effect is mostly driven but just one of the coexisting species (*Ochrobactrum* sp.), suggesting that previous reports on the positive relationship between species richness and niche exclusion is not a general effect, but simply the product of an increased likelihood of sampling at least one species with strong competitive abilities. These results may help to understand the inconsistent effects of interspecific competitors on diversification of resident species across different studies and suggest that the presence of niche-specific competitors represents a potential explanation.

However, on a broader scale, Bajić et al. make the case that the process of niche construction, by which organisms alter their environments creating novel evolutionary opportunities, plays a major role on how microbial communities assemble, diversify and evolve. Specifically, the authors argue that niche construction may provide support to genotypes that are not viable in the original niche, but that can act as “stepping-stones” to previously inaccessible regions of the genotypic space. However, a systematic, direct exploration of these possibilities is beyond current experimental capabilities. To address this issue, the authors point at the recent advances in genome-scale metabolic models (e.g., platforms such as “Computation of Microbial Ecosystems in Time and Space”—COMETS) that are now able to capture complex phenomena observed in microbial communities, such as niche construction. The authors suggest that these genome-scale metabolic models offer a unique opportunity to understand biological processes at lower levels of organization without isolating them from the eco-evolutionary processes in which they are embedded. Yet, while metabolic models can predict the release of some compounds, the origin of complex metabolic mixtures is still poorly understood within microbial communities.

Understanding how microbial diversity scales through space, between connected ecosystems, is a key question for microbial ecology. This gains additional relevance when considering the microbiota connectivity among humans and across the collection of ecosystems within the human digestive tract, which play a crucial role in human health (Donaldson et al., 2016). Species-area relationship (SAR) models have long been a key tool for ecologists to analyse the scaling of biodiversity across space. In their study, Chen et al. apply a recently developed extension of the classical SAR, named the diversity-area relationship (DAR) to understand how the human digestive tract (DT) microbiota biodiversity changes both within (across DT sites) and across human adults. Their analysis shows that the main differences between individuals in terms of microbial diversity were at the level of taxa presence/absence (i.e., species richness) rather than at the level of taxa abundance (e.g., Shannon entropy or Simpson index). When comparisons were made at the DT site level, differences between sites were maintained across diversity metrics and were clearly stronger than between individuals, as would be expected given the strong biological differences between DT sites. The study reveals how inter and intra-individual variation shapes microbial biodiversity in healthy adults and opens the possibility of using the diversity and the ecological structure of the microbiota as biomarkers of disease, rather than the presence of specific microbes.

The study of microbial ecology and evolution is currently largely dependent on *in silico* approaches. Comparative genome analyses are at the core of microbiome research and are crucial to decipher the evolutionary relationships between species, giving also clinical clues in case of bacterial pathogens. By comparing the genomes of 290 strains of *Clostridium perfringens*, Jaakkola et al. disclose two distinct sub-groups of *C. perfringens* strains with different virulence characteristics, spore heat resistance properties, and, presumably, ecological niche. The authors reveal that spore heat resistance of *C. perfringens* strains is likely affected by multiple genes and the capacity to produce heat-resistant spores has developed primarily within one subgroup of chromosomal enterotoxin-carrying strains. Studies based on comparative genomics are thus instrumental to elucidate the population structure and ecology of bacterial species and communities. Coupling taxonomic comparison with microbial function represents a further step in understanding microbiome evolution.

In this regard, Chen et al. examined 938 annotated water metagenomes obtained worldwide to investigate the connection between function and taxonomy, and to identify the key drivers of water metagenomes function or taxonomic composition at a global scale. The authors find that microbial functions are significantly correlated to taxonomy in aquatic ecosystems, with salinity being the predominant driver of microbial functional and taxonomic diversities. Classification into six water biomes resulted in greater variation in taxonomic compositions than functional profiles, highlighting significant functional redundancy at a global level.

Finally, Kumar et al. consider how environmental pressure shapes microbial evolution, by providing an extensive review on the mechanisms of marine fungi adaptation to climate change. The authors show that environmental stressors cause a direct alteration of cellular and molecular pathways in *Hortaea werneckii* and *Aspergillus terreus*, which ultimately leads to fungal adaptation to environmental change. The authors claim that understanding the mechanisms and the extent to which microbes adapt to environmental fluctuations, particularly in the era of climate change, is key to help researchers to develop transgenic organisms that are able to grow in such environments and whose flexibility will reduce the risk of climate change impacts on environments and organisms.

Altogether, these studies showcase the need for a multi-pronged approach to gain insights into the complex relationship among the environment, microbiota composition and evolutionary outcomes. Taken together, there is reason to hope that integrating different approaches (experimental evolution, comparative metagenomics, metabolic models and functional associations) will pave the way to a better understanding of microbial evolution. This will also allow predicting evolution in many important real life scenarios, such as antibiotic resistance evolution, human microbiome functioning under development, aging and disease, or soil microbiome stability and crop yields under accelerating rates of global change.

## Author contributions

All authors listed have made a substantial, direct, and intellectual contribution to the work and approved it for publication.

## Funding

AQ was supported by the Supporting Talent in Research @ University of Padua – STARS Grants 2019. AC was supported by the Spanish Ministry of Science, Innovation, and Universities (PID2019-110992GA-I00) and the regional government of Madrid (2019-T1/BIO-12882). RR was supported by the European Social Fund (ALT20-05-3559-FSE-000036) and the Fundação para a Ciência e a Tecnologia (FCT) based on RCM 23/8 March 2018. MM was supported by the Supporting Talents @Univesrity of Padua - STARS Grant 2017 and by the Italian Ministry for Universities and Research (MUR).

## Conflict of interest

The authors declare that the research was conducted in the absence of any commercial or financial relationships that could be construed as a potential conflict of interest.

## Publisher's note

All claims expressed in this article are solely those of the authors and do not necessarily represent those of their affiliated organizations, or those of the publisher, the editors and the reviewers. Any product that may be evaluated in this article, or claim that may be made by its manufacturer, is not guaranteed or endorsed by the publisher.
